# Inflammation and Renal Function after a Four-Year Follow-Up in Subjects with Unimpaired Glomerular Filtration Rate: Results from the Observational, Population-Based CARLA Cohort

**DOI:** 10.1371/journal.pone.0108427

**Published:** 2014-09-26

**Authors:** Daniel Medenwald, Matthias Girndt, Harald Loppnow, Alexander Kluttig, Sebastian Nuding, Daniel Tiller, Joachim J. Thiery, Karin H. Greiser, Johannes Haerting, Karl Werdan

**Affiliations:** 1 Institute of Medical Epidemiology, Biostatistics and Informatics, Martin-Luther-University Halle-Wittenberg, Halle/Saale, Germany; 2 Department of Internal Medicine II, Martin-Luther-University Halle-Wittenberg, Halle/Saale, Germany; 3 Department of Internal Medicine III, Martin-Luther-University Halle-Wittenberg, Halle/Saale, Germany; 4 Institute of Laboratory Medicine, Clinical Chemistry and Molecular Diagnostics, University of Leipzig, Leipzig, Germany; 5 German Cancer Research Centre, Division of Cancer Epidemiology, Heidelberg, Germany; Shanghai Institute of Hypertension, China

## Abstract

**Background:**

There is evidence that chronic inflammation is associated with the progression/development of chronic renal failure; however, relations in subjects with preserved renal function remain insufficiently understood.

**Objective:**

To examine the association of inflammation with the development of renal failure in a cohort of the elderly general population.

**Methods:**

After excluding subjects with reduced estimated glomerular filtration rate (eGFR<60 mL/min/1.73 m^2^) and missing data, the cohort incorporated 785 men and 659 women (aged 45–83 years). Follow-up was performed four years after baseline. Covariate adjusted linear and logistic regression models were used to assess the association of plasma/serum concentrations of soluble tumour necrosis factor receptor 1 (sTNF-R1), C-reactive protein (CRP), and interleukin 6 (IL-6) with change in eGFR/creatinine. The areas under the curve (AUCs) from receiver operating characteristics (ROCs) were estimated.

**Results:**

In adjusted models sTNF-R1 was distinctively associated with a decline in eGFR in men (0.6 mL/min/1.73 m^2^ per 100 pg/mL sTNF-R1; 95% CI: 0.4–0.8), but not in women. A similar association could not be found for CRP or IL-6. Estimates of sTNF-R1 in the cross-sectional analyses were similar between sexes, while CRP and IL-6 were not relevantly associated with eGFR/creatinine.

**Conclusion:**

In the elderly male general population with preserved renal function sTNF-R1 predicts the development of renal failure.

## Introduction

The role of inflammation in the development of diseases is of major research interest in almost all fields of medicine including nephrology. Recent studies showed an inverse relation of C-reactive protein (CRP) and renal function in chronic kidney disease [1] and diabetic patients [2], respectively. However, other studies failed to find a relevant association between CRP and kidney function in population based samples [3]. Furthermore, it was revealed that tumour necrosis factor α (TNF- α) and related pathways play an important role in the pathophysiology of kidney diseases [4], [2], [1]. In particular, the soluble tumour necrosis factor receptor type 1 (sTNF-R1) was found to correlate remarkably with Cystatin C in a cross-sectional analysis [4], [5]. The stability of sTNF-R1 makes it an easily assessable marker of the larger TNF system [6]. In a longitudinal analysis of patients with type 2 diabetes sTNF-R1 [7] and the second type of soluble tumour necrosis factor receptor (sTNF-R2) [7], [8] were associated with a decline in glomerular filtration rate (GFR), which was similar to findings in a population-based cohort [9]. Taking these previous findings into account, sTNF-R1 might serve as a promising biomarker for predicting a decline in renal function in asymptomatic persons in the general population. Shankar et al. [9] reported considerable longitudinal associations between inflammation parameters and progression of renal failure in a population-based collective. However, when subjects with reduced renal function were excluded from the analysis, only sTNF-R1 and Interleukin 6 (IL-6), but not CRP, remained positively associated with incident chronic kidney disease. Thus, a relevant amount of the described associations might be driven by the presence of chronic kidney disease, which could itself cause augmented plasma levels of inflammation parameters. Even more, the formula recently introduced by the Chronic Kidney Disease Epidemiology Collaboration (CKD-EPI formula) [10] estimates the GFR above values of 60 mL/min/1.73 m^2^ with less bias than the Modification of Diet in Renal Disease (MDRD) formula [11]. This enables us to perform analyses containing renal function on a continuous scale more accurately in subjects with an unimpaired GFR. Additionally, sex differences regarding the association between inflammation and renal function have only been insufficiently examined. Thus, it was the goal of the current population-based study to analyse associations of inflammation parameters including sTNF-R1 with alterations of renal function in elderly men and women after excluding subjects with reduced renal function at baseline.

## Methods

### Study cohort

We used data from the ***CAR*** *dio-vascular Disease,* ***L*** *iving and* ***A*** *geing in Halle* study (CARLA study), which is a prospective population-based cohort study of the elderly general population of the city of Halle in eastern Germany [12], [13]. The CARLA cohort comprises 1,779 participants aged 45–83 years at baseline (812 women, 967 men). The baseline examination took place between December 2002 and January 2006. A multi-step recruitment strategy aimed to achieve a high response rate. The percentage final response after subtracting exclusions (individuals who were deceased prior to the invitation, had moved away, or were unable to participate due to illness) was 64.1%. The first four-year follow-up examination was performed from March 2007 until March 2010. The net sample (after the exclusion of deceased or non-responding people) then comprised 1,436 subjects (86% response), consisting of 790 men and 646 women aged between 50 and 87 years. The study participants underwent a detailed medical examination and a standardized, computer-assisted interview that collected information on socio-demographic and socioeconomic variables, behavioural, biomedical, and psychosocial factors, medical history, and the use of medication within the preceding seven days. Medication was automatically coded according to the Anatomical Therapeutic Chemical Classification System (ATC code). Additionally, an analysis of non-respondents was performed in order to assess non-response bias by obtaining information about prevalent diseases, and selected behavioural and sociodemographic factors. A more comprehensive account of the CARLA study can be found in Greiser et al. [12]. The study was approved by the Ethics Committee of the Medical Faculty of the Martin-Luther-University Halle-Wittenberg and by the State Data Privacy Commissioner of Saxony-Anhalt and conformed to the principles outlined in the Declaration of Helsinki [14]. All participants gave written informed consent.

We excluded subjects with missing values for the baseline parameters of creatinine, sTNF-R1, hsCRP or Il-6 (n = 195). Additionally, in the longitudinal analyses subjects with missing creatinine (baseline and follow-up) values were not considered. Furthermore, subjects with an estimated glomerular filtration rate (eGFR) below 60 mL/min/1.73 m^2^ at baseline (76 men and 64 women) were excluded from the analysis. Thus, the total study population comprised of 785 men (46–83 years) and 659 women (45–83 years) at baseline, while 645 men (50–87 years) and 535 women (50–87 years) were considered in the longitudinal analyses. The eGFR was calculated using the CKD-EPI formula [10], as the generally applied MDRD formula [11] was found to have a considerable fluctuation and bias above a GFR of 60 mL/min/1.73 m^2^ in the general population [15]. Additionally, in the collective used to establish the CKD-EPI formula people older than 75 years seemed to be underrepresented [10] weakening its generalizability in the elderly. Thus, we conducted a sensitivity analysis whereby all subjects older than 71 years at baseline were excluded (detailed results given in the appendix).

### Laboratory measurements

Blood samples were taken after a supine rest of 30 minutes. The inflammation parameters of sTNF-R1 and IL-6 were analysed by the Department of Medicine III, University Clinics Halle (Saale). After a 10-min centrifugation (20°C, 1,500 rpm, Acc = 9, Dcc = 3), the plasma was collected and stored at −80°C. The cytokines were determined using commercially available sandwich enzyme-linked immunosorbent assays (ELISAs: IL-6, Opteia, BD Biosciences, Heidelberg, Germany; TNF-R1, Boehringer Mannheim, Mannheim, Germany).

The determination of CRP and creatinine was undertaken by the Institute of Laboratory Medicine, Clinical Chemistry and Molecular Diagnostics at Leipzig University Clinics. The laboratory has been accredited according to the accreditation norms ISO 15180 and ISO 17025. Serum levels of high-sensitivity CRP (hsCRP) were measured using a high-sensitivity immunoturbidimetric method (CRP [Latex] HS, Roche, Mannheim, Germany) on a Hitachi autoanalyser (Roche Diagnostics, Mannheim, Germany).

At baseline and at follow-up serum creatinine was determined using an enzymatic method (Creatinine Plus for Roche Hitachi cobas, Roche Diagnostics, Mannheim, Germany with calibrator f.a.s, Roche). Referring to the CKD-EPI formula, it was found that enzymatic methods have an adequate agreement with the IDMS-traceable Jaffé method in healthy subjects, but perform better in diabetic patients, which underscores its usefulness in population-based cohorts [16].

### Statistical analysis

All analyses were performed separately for men and women. Descriptive results are displayed as geometric means with their respective 95% confidence intervals (CI).

We used multiple linear regression models to analyse the relationship between inflammation and renal function assessed via eGFR and creatinine, respectively. The regression models were adjusted for age, body mass index (BMI), HbA1c, low-density lipoprotein (LDL), high-density lipoprotein (HDL), baseline diastolic and systolic blood pressure and baseline to follow-up change in the longitudinal analyses, number of cigarettes/cigars/pipes smoked, presence of cardio-vascular diseases, regular intake of anti-diabetic (Anatomical Therapeutic Chemical Classification [ATC]: A10) and anti-hypertensive medication (ATC: C02/C03/C07/C08/C09), while users were coded as “1” and non-users coded as “0”. In the longitudinal regression analyses, we used the baseline to follow-up differences in eGFR and creatinine, respectively, as outcome without consideration of baseline eGFR values as a further covariate [17]. The idea beyond such a longitudinal analysis is to check for associations between a change in one parameter over time (from baseline to follow-up) depending on an explanatory variable, that influences the course of the dependent parameter. Finally, the adequacy of the considered regression models was assumed when the residuals were normally distributed, which was tested via Q-Q plot and Cook’s distance, which was required to be below one. In univariate and covariate adjusted linear regression models squared partial Pearson correlation coefficients (*r^2^*) were additionally calculated.

We performed logistic regression analyses to estimate the odds of developing reduced renal function (eGFR<60 mL/min/1.73 m^2^) from baseline to follow-up adjusting for the previously mentioned covariates, and calculated the respective receiver operating characteristics (ROCs). Here, in order to make effect sizes comparable, odds ratios (ORs) refer to an increase of one standard deviation in the log-transformed inflammation parameter.

In the ROC graphs we defined the optimal cut off by using the minimal d-distance 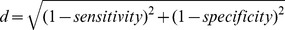.

The limit of statistical significance was assumed at an α of 5%. All statistical analyses and data management were performed using SAS, Version 9.3 (SAS Inc., Cary, NC, USA).

## Results

### Study population

In our study population we found higher values of sTNF-R1 in men compared to females (Table 1), which also held true for creatinine and eGFR. Additional parameters taken as covariates into account had almost identical mean values between the two sexes. From baseline to follow-up we found an increase in creatinine and thus a decline in eGFR across the whole population. Again, additional parameters such as BMI, LDL, HDL, and HbA1c changed only slightly, in contrast to systolic and diastolic blood pressure where a baseline to follow-up decrease was seen (Table 1). Sixty men and 44 women developed a renal impairment with an eGFR below 60 mL/min/1.73 m^2^.

**Table 1:** Subject characteristics.

	Baseline (Mean with 95% CI)				Follow-up (Mean with 95% CI)		
	Men (N = 785)	Women (N = 659)	p†		Men (N = 645)	Women (N = 535)	p†
sTNF-R1 (pg/mL)	1127.2 [1100.0, 1155.1]	1025.3 [999.1, 1052.2]	<.0001				
hsCRP (mg/L)	1.7 [1.6, 1.9]	1.8 [1.7, 2.0]	0.2054		1.9 (1.7, 2.0)**	1. 9 (1.7, 2.1)*	0.7757
IL-6 (pg/mL)	2.0 [1.8, 2.2]	1.8 [1.7, 2.0]	0.1727				
Creatinine (µmol/L)	76.6 [75.8, 77.4]	62.3 [61.5, 63.0]	<.0001		81.2 [80.0, 82.4]**	66.6 [65.5, 67.6]**	<.0001
eGFR (mL/min/1.73 m^2^)	88.2 [87.3, 89.1]	87.3 [86.3, 88.2]	0.1881		81.7 [80.3, 83.0]**	80.2 [78.8, 81.5]**	0.1215
Age (years)	63.3 [62.6, 64.0]	62.1 [61.4, 62.8]	0.0146		66.4 [65.6, 67.1]**	65.4 [64.6, 66.1]**	0.0617
BMI (kg/m^2^)	27.9 [27.6, 28.1]	27.9 [27.5, 28.3]	0.8759		27.9 [27.6, 28.2]*	28.0 [27.6, 28.4]**	0.8979
Dia. BP (mmHg)	85.3 [84.5, 86.1]	82.6 [81.8, 83.4]	<.0001		80.3 [79.5, 81.1]**	78.8 [78.0, 79.6]**	0.0096
Sys. BP (mmHg)	144.3 [143, 145.7]	139.5 [137.9, 141.2]	<.0001		137.6 [136.2, 139.1]**	134.1 [132.5, 135.8]**	0.0016
LDL (mmol/L)	3.0 [3.0, 3.1]	3.3 [3.2, 3.3]	<.0001		3.0 [2.9, 3.1]*	3.3 [3.2, 3.4]	<.0001
HDL (mmol/L)	1.2 [1.2, 1.3]	1.5 [1.5, 1.5]	<.0001		1.2 [1.2, 1.2]**	1.5 [1.4, 1.5]**	<.0001
HbA1c (%)	5.7 [5.6, 5.7]	5.7 [5.6, 5.7]	0.3443		5.8 [5.8, 5.9]**	5.8 [5.7, 5.9]**	0.4922
Daily cigarettes in	15 (N = 200)	12 (N = 102)	0.2857		15 (N = 129)	10 (N = 68)	0.6530
Smokers (Median)							
**Number of subjects**							
Cardiovasc. diseases	122	46		<.0001	139*	55*	<.0001
PAD	26	5		0.0009	51*	22*	0.0076
Anemia	5	7		0.3989			
eGFR<60 mL/min/1.73 m^2^	–	–			60	44	0.4753
Anti-hypertensive	410	346		0.9578	399*	341*	0.7512
medication							
Anti-diabetic	94	68		0.3573	91	64	0.2294
medication							

Baseline and follow-up means, displayed as geometric means with respective 95% confidence limits.

Abbreviations: sTNF-R1: Soluble tumour necrosis factor-α receptor 1; hsCRP: High-sensitivity C-reactive protein; IL-6: Interleukin 6; eGFR: estimated glomerular filtration rate; BMI: Body mass index; Dia. BP: Diastolic blood pressure; Sys. BP: Systolic blood pressure; LDL: Low-density lipoprotein; HDL: High-density lipoprotein; PAD: Peripheral arterial disease.

† p-values for mean differences between men and women (using T-test) or differences in numbers between men and women (using Fisher’s exact test); ** indicates p-values <0.0001 for baseline to follow-up change; * indicates p-values <0.05 for baseline to follow-up change.

### Cross-sectional analysis

In the cross-sectional analysis we found a considerable association of sTNF-R1 and creatinine in the univariate regression model and after adjustment for covariates which appeared to be comparable between men and women (Table 2, 3). With respect to eGFR there was an apparent negative association in both sexes after covariates were taken into account. Partial correlation coefficients of the association of sTNF-R1 with renal parameters were slightly stronger in men (around 6%, Table 2) than in women (around 4%, Table 3) when confounders were considered. We found no association of hsCRP and IL-6 with either eGFR or creatinine in men or women (Table 2, 3).

**Table 2:** Cross-sectional and longitudinal regression analyses in men: association of inflammation parameters with GFR/creatinine.

Cross-sectional analysis							
Continuous Outcome		eGFR (95% CI)[mL/min/1.73 m^2^]	p	Partial correlationcoefficient	Creatinine (95% CI)[mmol/L]	p	Partial correlationcoefficient
sTNF-R1 (100 pg/mL)	unadj.	−1.1 (−1.3, −1.0)	<.0001	0.173	0.8 (0.6, 0.9)	<.0001	0.094
	adj.	−0.6 (−0.7, −0.4)	<.0001	0.064	0.7 (0.5, 0.9)	<.0001	0.069
hsCRP (10 mg/L)	unadj.	−1.3 (−3.4, 0.7)	0.2083	0.002	1.1 (−0.8, 3.0)	0.2478	0.002
	adj.	−1.2 (−2.7, 0.3)	0.1197	0.003	1.4 (−0.5, 3.3)	0.1386	0.003
IL-6 (10 pg/mL)	unadj.	−0.3 (−1.1, 0.6)	0.8398	<0.001	0.0 (−0.4, 0.4)	0.9652	<0.001
	adj.	0.3 (−0.4, 0.9)	0.4005	<0.001	0.0 (−0.4, 0.4)	0.8398	<0.001
**Longitudinal analysis (change in eGFR/Creatinine)**							
**Continuous Outcome**		**eGFR (95% CI)** **[mL/min/1.73** **m^2^]**	**p**	**Partial correlation** **coefficient**	**Creatinine (95% CI)** **[mmol/L]**	**p**	**Partial correlation** **coefficient**
sTNF-R1 (100 pg/mL)	unadj.	−0.7 (−0.9, −0.5)	<.0001	0.081	1.2 (0.9, 1.4)	<.0001	0.107
	adj.	−0.6 (−0.8, −0.4)	<.0001	0.053	1.0 (0.7, 1.3)	<.0001	0.072
hsCRP (10 mg/L)	unadj.	−0.6 (−2.5, 1.2)	0.4973	0.001	0.5 (−2.0, 2.9)	0.7112	<0.001
	adj.	−0.4 (−2.2, 1.5)	0.6848	<0.001	0.3 (−2.2, 2.7)	0.8327	<0.001
IL-6 (10 pg/mL)	unadj.	−0.4 (−1.1, 0.3)	0.2801	0.002	0.8 (−0.2, 1.8)	0.1124	0.004
	adj.	−0.2 (−0.9, 0.5)	0.5367	<0.001	0.6 (−0.4, 1.6)	0.2352	0.003
**Incident Renal Failure:** **<60 mL/min/1.73** **m^2^**		Odds Ratio (95% CI)*	p	Optimal Cut Off			
				(Sensitivity/specificity)			
sTNF-R1	unadj.	3.4 (2.4, 4.9)	<.0001	1285.3 (76.7/75.4)			
	adj.	2.6 (1.7, 4.1)	<.0001				
hsCRP	unadj.	1.3 (1.0, 1.7)	0.0651	1.57 (63.3/52.2)			
	adj.	1.2 (0.9, 1.7)	0.2651				
IL-6	unadj.	1.1 (0.9, 1.4)	0.4680	1.77 (55.0/52.8)			
	adj.	1.1 (0.8, 1.4)	0.7501				

unadj. = unadjusted estimates; adj. = estimates adjusted for age, body mass index (BMI), HbA1c, low-density lipoprotein (LDL), high-density lipoprotein (HDL), baseline diastolic and systolic blood pressure and baseline to follow-up change in the longitudinal analyses, number of cigarettes/cigars/pipes smoked, presence of cardio-vascular diseases, regular intake of anti-diabetic (Anatomical Therapeutic Chemical Classification [ATC]: A10) and anti-hypertensive medication (ATC: C02/C03/C07/C08/C09); users coded as “1”, non-users coded as “0”. GFR estimated by means of CKD-EPI formula^10^. Effect estimates with 95% confidence intervals are displayed.

*Effect estimates refer to an increase of one standard deviation in the log-transformed inflammation parameter.

Abbreviations: sTNF-R1: Soluble tumour necrosis factor-α receptor 1; hsCRP: High-sensitivity C-reactive protein; IL-6: Interleukin 6; eGFR: estimated glomerular filtration rate.

**Table 3:** Cross-sectional and longitudinal regression analyses in women: association of inflammation parameters with GFR/creatinine.

Cross-sectional analysis							
Continuous Scale		eGFR (95% CI)[mL/min/1.73 m^2^]	p	Partial correlationcoefficient	Creatinine (95% CI)[mmol/L]	p	Partial correlationcoefficient
sTNF-R1 (100 pg/mL)	unadj.	−1.2 (−1.4, −0.9)	<.0001	0.145	0.6 (0.4, 0.7)	<.0001	0.059
	adj.	−0.6 (−0.8, −0.4)	<.0001	0.044	0.5 (0.3, 0.7)	<.0001	0.043
hsCRP (10 mg/L)	unadj.	1.0 (−1.2, 3.2)	0.3782	0.001	0.7 (−2.4, 1.0)	0.4380	<0.001
	adj.	0.7 (−1.2, 2.7)	0.4740	<0.001	−0.7 (−2.5, 1.1)	0.4539	<0.001
IL-6 (10 pg/mL)	unadj.	−0.1 (−0.8, 0.6)	0.7509	<0.001	−0.1 (−0.3, 0.1)	0.3108	0.002
	adj.	0.1 [−0.5, 0.7]	0.7114	<0.001	−0.1 [−0.3, 0.1]	0.2745	0.002
**Longitudinal analysis (change in eGFR/Creatinine)**							
**Continuous Scale**		**eGFR (95% CI)** **[mL/min/1.73** **m^2^]**	**p**	**Partial correlation** **coefficient**	**Creatinine (95% CI)** **[mmol/L]**	**p**	**Partial correlation** **coefficient**
sTNF-R1 (100 pg/mL)	unadj.	−0.2 (−0.4, 0.0)	0.0946	0.005	0.3 (0.0, 0.5)	0.0217	0.010
	adj.	−0.1 (−0.3, 0.2)	0.4763	0.001	0.1 (−0.1, 0.4)	0.3233	0.002
hsCRP (10 mg/L)	unadj.	−1.3 (−3.7, 1.1)	0.3010	0.002	1.6 (−1.0, 4.2)	0.2350	0.003
	adj.	−0.7 (−3.3, 1.9)	0.5989	0.001	1.4 (−1.4, 4.1)	0.3237	0.002
IL-6 (10 pg/mL)	unadj.	−1.4 (−2.5, −0.3)	0.0147	0.011	1.2 (0.0, 2.4)	0.0480	0.007
	adj.	−1.4 (−2.6, −0.3)	0.0123	0.011	1.2 (0.0, 2.4)	0.0530	0.007
**Incident Renal Failure: <60** **mL/min/1.73 m^2^**		Odds Ratio (95% CI)*	p	Optimal Cut Off			
				(Sensitivity/specificity)			
sTNF-R1	unadj.	2.5 (1.7, 3.5)	<.0001	1142.0 (70.5/71.5)			
	adj.	2.0 (1.3, 3.0)	0.0014				
hsCRP	unadj.	1.4 (1.0, 2.0)	0.0365	2.13 (59.0/58.2)			
	adj.	1.5 (1.0, 2.2)	0.0703				
IL-6	unadj.	1.1 (0.8, 1.4)	0.7582	1.33 (72.7/39.5)			
	adj.	1.0 (0.7, 1.4)	0.9592				

unadj. = unadjusted estimates; CI = confidence limit; adj. = estimates adjusted for age, body mass index (BMI), HbA1c, low-density lipoprotein (LDL), high-density lipoprotein (HDL), baseline diastolic and systolic blood pressure and baseline to follow-up change in the longitudinal analyses, number of cigarettes/cigars/pipes smoked, presence of cardio-vascular diseases, regular intake of anti-diabetic (Anatomical Therapeutic Chemical Classification [ATC]: A10) and anti-hypertensive medication (ATC: C02/C03/C07/C08/C09); users coded as “1”, non-users coded as “0”. GFR estimated by means of CKD-EPI formula^10^. Effect estimates with 95% confidence intervals are displayed.

*Effect estimates refer to an increase of one standard deviation in the log-transformed inflammation parameter.

Abbreviations: sTNF-R1: Soluble tumour necrosis factor-α receptor 1; hsCRP: High-sensitivity C-reactive protein; IL-6: Interleukin 6; eGFR: estimated glomerular filtration rate.

### Longitudinal analysis

#### Change in serum creatinine and eGFR

**Figure 1:**
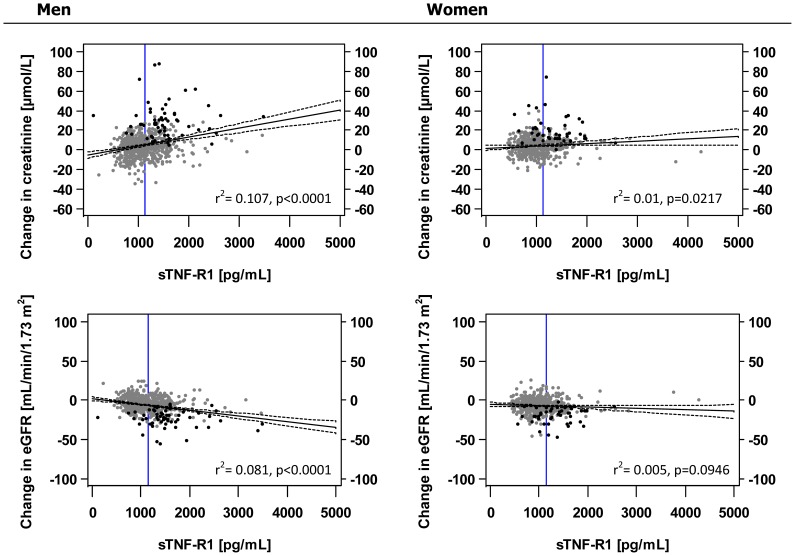
sTNF-R1 and change in eGFR/creatinine from baseline to follow-up. Black line: regression line with corresponding 95% (dashed line); vertical blue line: optimal cut off; black dots: subjects with eGFR<60 mL/min/1.73 m^2^ at follow-up; grey dots: subjects with eGFR>60 mil/min at follow-up. Abbreviations: sTNF-R1: Soluble tumour necrosis factor-α receptor 1, eGFR: estimated glomerular filtration rate.

**Figure 2:**
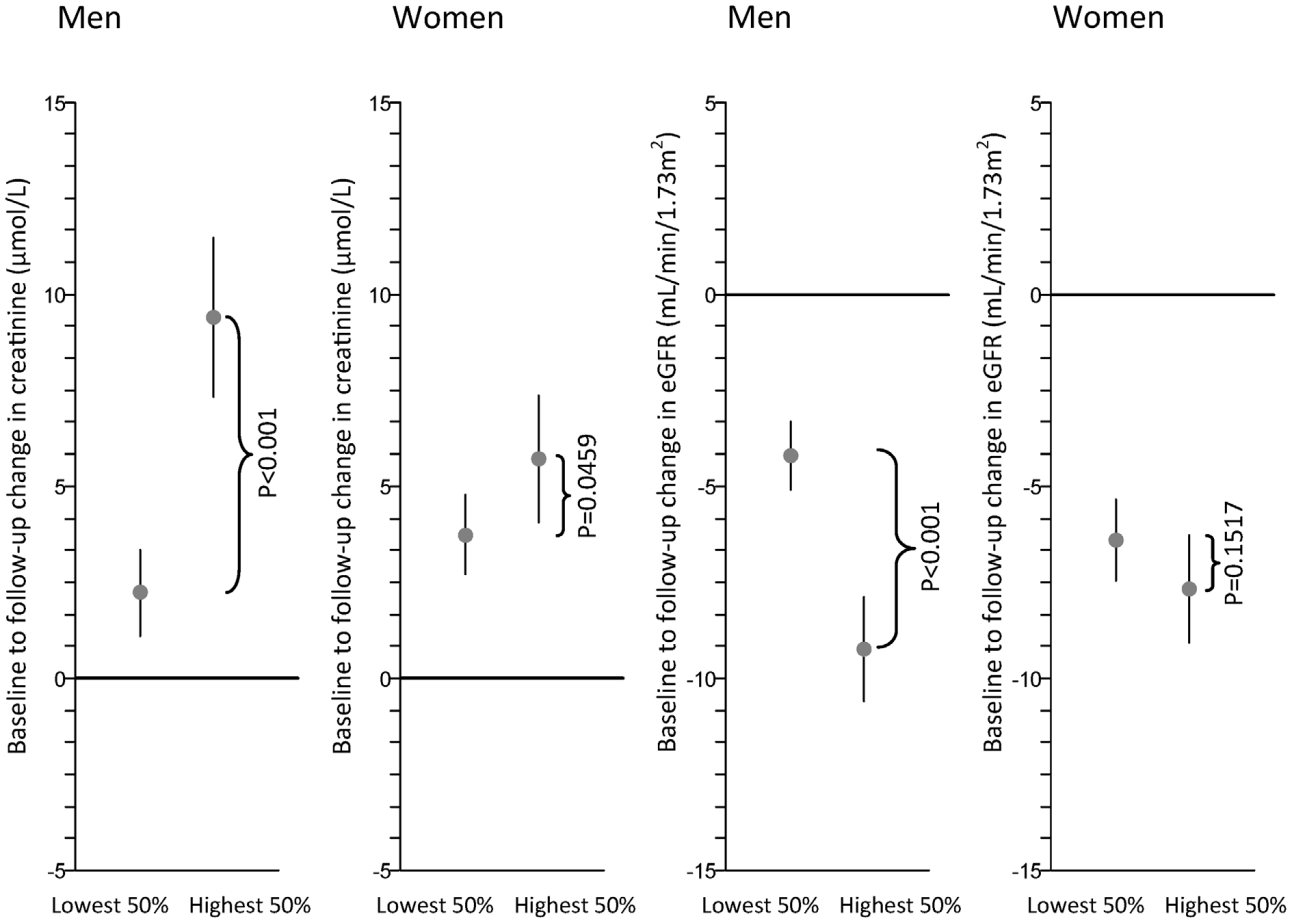
Change in mean eGFR/creatinine plasma level in subjects with the 50% lowest and 50% highest sTNF-R1 plasma levels at baseline. Abbreviations: sTNF-R1: Soluble tumour necrosis factor-α receptor 1, eGFR: estimated glomerular filtration rate.

Using the difference in creatinine between baseline and follow-up as the dependent variable, we found a strong association between sTNF-R1 and this outcome both in the univariate and adjusted regression model in men, which was estimated to increase by 1 µmol/L per 100 pg/mL increase in sTNF-R1 (95% CI: 0.7–1.3). This was in contrast to women, where effect estimates and partial correlation coefficients appeared to be smaller (β = 0.1; 95% CI: −0.1–0.4) and, thus, were not significant after covariate adjustment. Analysing eGFR, we again observed a remarkable negative association of sTNF-R1 with a baseline to follow-up difference in men (0.6; 95% CI: 0.4–0.8); however, it was substantially weaker in women (Table 2, 3; Figure 1). Coming to the partial correlation, roughly 5% of the variance in baseline to follow-up change in eGFR could be explained by the direct effect of sTNF-R1 (Table 2) in men. Similarly, men with the 50% highest values of sTNF-R1 showed a strong decline in eGFR and an increase in creatinine, which is not true for female subjects (Figure 2). The relevance of different effect sizes between men and women is underpinned by a significant, multiplicative interaction of sex and sTNF-R1 when both creatinine (p<0.0001, results not shown) and eGFR (p = 0.0001, results not shown) were the outcome. Analysing the predictive value of hsCRP and IL-6, respectively, we could not reveal any relevant effect of either parameter on the progression of renal function on a continuous scale.

#### Incident Renal Failure

**Figure 3:**
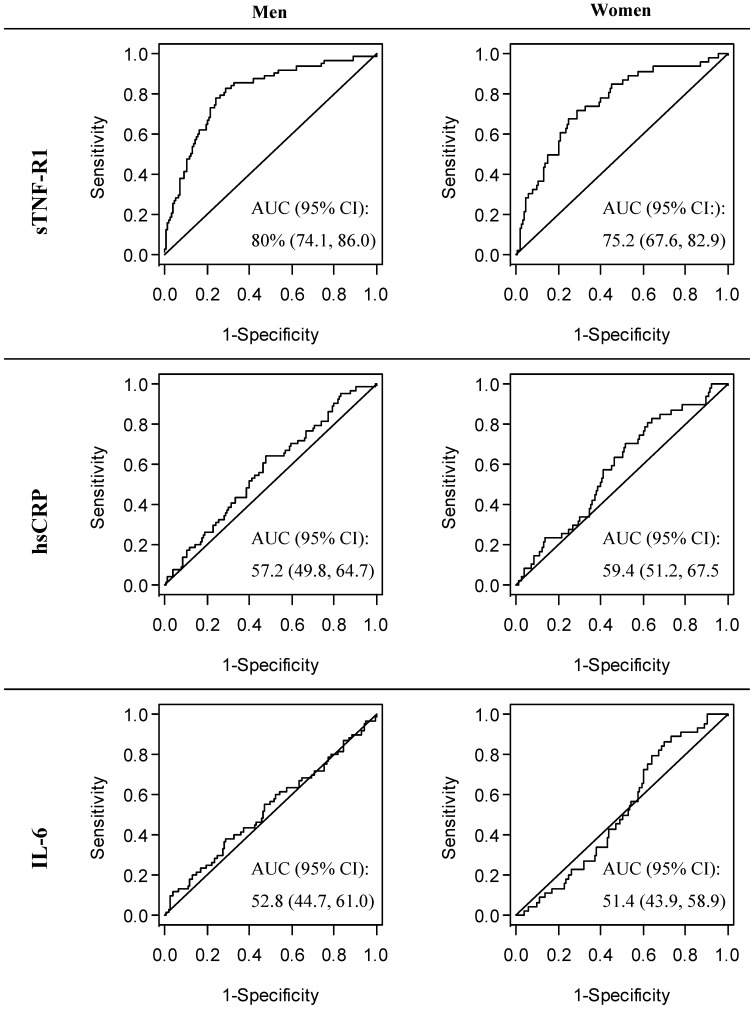
ROC curves and respective AUCs [95% confidence intervals]. Abbreviations: sTNF-R1: Soluble tumour necrosis factor-α receptor 1; hsCRP: High- sensitivity C-reactive protein; IL-6: Interleukin 6; CI: confidence interval.

When we examined the prognostic value of sTNF-R1 in forecasting renal impairment (eGFR<60 mL/min/1.73 m2), the covariate adjusted OR was calculated to be 2.6 (95% CI: 1.7–4.1) in men and 2.0 (95% CI: 1.3–3.0) in women, respectively (Table 2, 3). Using sTNF-R1 as the only predictive parameter in a prognostic model, we found an AUC of 80.0% (95% CI: 74.1%–86.0%) in men and an AUC of 75.2% (95% CI 67.6%–82.9%) in women (Figure 3). The optimal cut off was determined at a value of 1285.3 pg/nL in men and 1142.0 pg/nL in women (Table 2, 3). Taking these cut offs as a basis, the sensitivity and specificity of sTNF-R1 was 76.7% and 75.4%, respectively, in men. In women the optimal cut off was related to a sensitivity of 70.5% and a specificity of 71.5%. The beta coefficients in the analysis of hsCRP were considerably weaker than those calculated in the analysis of sTNF-R1. In men we found an AUC of 57.2% (95% CI: 49.8%–64.7%) and an optimal cut off at 1.57 mg/L accompanied by a sensitivity and specificity below the one found in the analysis of sTNF-R1. In women the AUC of hsCRP again was estimated to be marginally lower than in men. For IL-6 we failed to reveal a clear association pattern with renal failure as in the case of sTNF-R1 and hsCRP in either sex (Table 2, 3); here we estimated similar AUCs as in the case of hsCRP for both sexes, which is also true for the sensitivity and specificity at the optimal cut offs (Table 2, 3).

### Sensitivity analysis

When we used the MDRD formula to estimate the GFR, effect estimates increased partly in both the cross-sectional and longitudinal analyses compared to the GFR calculation by using the CKD-EPI formula (Tables S1, S2 in File S1). After excluding subjects over 71 years of age at baseline, the estimates from the cross-sectional analysis decreased, while the results of the longitudinal analyses were virtually unaltered (Tables S3, S4 in File S1).

## Discussion

To summarize our findings, we disclosed a notable association between sTNF-R1 and reduced kidney function independent of considered covariates in cross-sectional and longitudinal analyses (here in men), with similar estimates in both sexes in the former case. However, a similar predictive value was not contributed by IL-6 or hsCRP in terms of continuous associations. In the logistic regression we found a significant but weak relation of IL-6 and hsCRP with the outcome of reduced kidney function, despite a remarkable association in the case of sTNF-R1. As sTNF-R1 was related to a longitudinal change in renal function in men, rather than with the mere parameters in a cross-sectional way, a reversal causation can be excluded. Similar to our findings, Miyazawa et al. reported an association of sTNF-R1 with kidney function; however, their collective was based on diabetic patients [7]. Using a population-based sample, a more recent study by Shankar et al. [9] reported a considerable association of the type 2 TNF-α receptor with the development of kidney failure after a 15-year follow-up. On a cellular level, both TNF-α and sTNF-R1 are intertwined: sTNF-R1 is released from its membrane bound form due to the effect of certain stimuli including TNF-α [18]. TNF-α initiates several pathways in humans affecting cell metabolism and apoptosis through its membranous receptors [19], [20]. Lin et al. regarded the type 2 TNF-α receptor as a surrogate of TNF-α activation, and again found a significant association of this parameter with kidney function. As Clendenen et al. [21] disclosed, both are correlated with each other, but also with further inflammation parameters. Several mechanisms regarding the effect of TNF-α on kidneys have been proposed: Most importantly, TNF-α has been found to cause a reduction in kidney function via complex pathways (mainly due to its cytotoxicity towards glomerular, mesangial, and epithelial cells) [22], including renal fibrosis, where mechanisms such as an activation of nuclear factor κB regulating cell adhesion molecules, a promotion of TGF-β_1_ and an altered release of nitric oxide are essential [23]. In addition, TNF-α plays a key role in mesangial cells proliferation and collagen synthesis [24]. TNF-α exhibits additional renal damage by being related to the blood pressure increasing effect of angiotensin II [20], [25]. Through a direct effect and via nitrate/nitrite, TNF-α has a considerable effect on ion (sodium/potassium) and water absorption in kidneys, which might also contribute to changes in renal function [20]. Apart from the mentioned, further signal transducing proteins such as TNF-related apoptosis-inducing ligand (TRAIL) have been proposed to induce renal apoptosis in non-autoimmune diabetic patients [26], [27]. Thus, TNF-α by its transmembrane receptors seems to induce a large variety of processes that have the potential to worsen renal function.

Our results regarding hsCRP are in contrast to those of Gupta et al. [1], which might be due to their collective of chronic kidney disease patients. Focusing on a sample of the general population, Pruijm et al. [3] could not confirm a relevant association of hsCRP with renal function. However, the same study found an association of TNF-α with eGFR, which is consistent with our finding regarding sTNF-R1.

The disclosed sex differences are remarkable; however, sex specific analyses have rarely been conducted. Previous research disclosed important sex-specific effects of several hormones including angiotensin II, endothelin and sex hormones in renal damage whereby a protective effect of estrogens was proposed [28]. In contrast, a study by Pai et al. [29] revealed a stronger effect of sTNF-R1 on cardio-vascular events in women. In our study two effects might account for most of the observed sex differences: 1) A protective effect of female hormones or an adverse effect due to testosterone on renal damage induced by TNF-α. Indeed, it was shown that testosterone promotes TNF-α induced obstructive renal injuries by inducing proapoptotic and profibrotic pathways [30] and has a pro-inflammatory effect on the endothelium [31]. This is in line with our findings as men showed a higher plasma level of sTNF-R1 than women and thus might suffer from a dose-dependent TNF-α effect on renal function. The roughly 10% higher values in male subjects compared to females is of similar magnitude as a decrease in renal function was explainable by TNF-α after a four-year follow-up. 2) As the effect estimates were similar in men and women in the cross-sectional analyses a time dependent effect might be likely, i.e. it might take much shorter or longer for TNF-α to have a relevant effect on kidneys than our four-year follow-up. Apart from the direct effect of sex hormones on TNF-α production, it was shown that male neutrophils respond with a higher production of TNF-α after stimulation with lipopolysaccharides and Interferon-γ [32].

The effect estimates in our study regarding IL-6 are only slightly smaller than in the mentioned study by Shankar et al. [9]. Possible differences might be mainly due to the longer time span between baseline and follow-up of 15 years in their study. Nevertheless, it would be interesting to know the effect estimates in their study after exposure and outcome variables were accounted for in continuous terms. The slightly larger effect estimates in the analyses using the MDRD formula might reflect the previously mentioned bias of the MDRD formula in higher GFRs [10].

### Limitations

One major limitation of our study was the inclusion of only a single follow-up after four years; shorter or longer repetitive intervals might disclose further time effects and the temporal stability of the observed relations. With respect to our study collective from the elderly general population, we were not able to assess inflammation effects in younger subjects. Furthermore, the GFR was only estimated by means of plasma creatinine based formulae; however, further parameters such as Cystatin C or estimation of the GFR using 24 hours urine collections might give more accurate estimates of renal function. Thus, results should be interpreted with this limitation of an indirect approach to quantify renal function. Future studies using a direct method are needed to confirm our findings. In the current study only analyses of three inflammation parameters were conducted. However, respecting complex interactions between inflammation parameters, further inflammatory markers might reveal additional insights in the pathogenesis and determination of renal function. Even more, we are not able to provide data on the association of inflammation with urine biomarkers such as albumin, which might be related to structural impairments due to chronic inflammation.

In conclusion, we found longitudinal effects of sTNF-R1 on renal function in men, which were noticeably weaker in women. Thus, our results partly confirm the findings of Shankar et al. [9]; however, further studies focusing on time-dependent sex differences in inflammation effects are required in order to gain further insights into pathophysiological mechanisms.

## Supporting Information

File S1 Contains Table S1, Cross-sectional and longitudinal linear regression analysis in men: association of inflammation parameters with GFR/creatinine. Glomerular filtration rate estimated by means of MDRD formula. (effect estimates with 95% confidence intervals). unadj. = unadjusted estimates; adj. = estimates adjusted for age, body mass index (BMI), HbA1c, low-density lipoprotein (LDL), high-density lipoprotein (HDL), baseline diastolic and systolic blood pressure and baseline to follow-up change in the longitudinal analyses, number of cigarettes/cigars/pipes smoked, presence of cardio-vascular diseases, regular intake of anti-diabetic (Anatomical Therapeutic Chemical Classification [ATC]: A10) and anti-hypertensive medication (ATC: C02/C03/C07/C08/C09); users coded as “1”, non-users coded as “0”. Abbreviations: sTNF-R1: Soluble tumour necrosis factor-α receptor 1; hsCRP: High-sensitivity C-reactive protein; IL-6: Interleukin 6; eGFR: estimated glomerular filtration rate. Table S2, Cross-sectional and longitudinal linear regression analysis in women: association of inflammation parameters with GFR/creatinine. Glomerular filtration rate estimated by means of MDRD formula. (effect estimates with 95% confidence intervals). unadj. = unadjusted estimates; adj. = estimates adjusted for age, body mass index (BMI), HbA1c, low-density lipoprotein (LDL), high-density lipoprotein (HDL), baseline diastolic and systolic blood pressure and baseline to follow-up change in the longitudinal analyses, number of cigarettes/cigars/pipes smoked, presence of cardio-vascular diseases, regular intake of anti-diabetic (Anatomical Therapeutic Chemical Classification [ATC]: A10) and anti-hypertensive medication (ATC: C02/C03/C07/C08/C09); users coded as “1”, non-users coded as “0”. Abbreviations: sTNF-R1: Soluble tumour necrosis factor-α receptor 1; hsCRP: High-sensitivity C-reactive protein; IL-6: Interleukin 6; eGFR: estimated glomerular filtration rate. Table S3, Cross-sectional and longitudinal linear regression analysis in men: association of inflammation parameters with GFR/creatinine after exclusion of subjects older than 71 years at baseline. Glomerular filtration rate estimated by means of CKD-EPI formula. (effect estimates with 95% confidence intervals). unadj. = unadjusted estimates; adj. = estimates adjusted for age, body mass index (BMI), HbA1c, low-density lipoprotein (LDL), high-density lipoprotein (HDL), baseline diastolic and systolic blood pressure and baseline to follow-up change in the longitudinal analyses, number of cigarettes/cigars/pipes smoked, presence of cardio-vascular diseases, regular intake of anti-diabetic (Anatomical Therapeutic Chemical Classification [ATC]: A10) and anti-hypertensive medication (ATC: C02/C03/C07/C08/C09); users coded as “1”, non-users coded as “0”. Abbreviations: sTNF-R1: Soluble tumour necrosis factor-α receptor 1; hsCRP: High-sensitivity C-reactive protein; IL-6: Interleukin 6; eGFR: estimated glomerular filtration rate. Table S4, Cross-sectional and longitudinal linear regression analysis in women: association of inflammation parameters with GFR/creatinine after exclusion of subjects older than 71 years at baseline. Glomerular filtration rate estimated by means of CKD-EPI formula. (effect estimates with 95% confidence intervals). unadj. = unadjusted estimates; adj. = estimates adjusted for age, body mass index (BMI), HbA1c, low-density lipoprotein (LDL), high-density lipoprotein (HDL), baseline diastolic and systolic blood pressure and baseline to follow-up change in the longitudinal analyses, number of cigarettes/cigars/pipes smoked, presence of cardio-vascular diseases, regular intake of anti-diabetic (Anatomical Therapeutic Chemical Classification [ATC]: A10) and anti-hypertensive medication (ATC: C02/C03/C07/C08/C09); users coded as “1”, non-users coded as “0”. Abbreviations: sTNF-R1: Soluble tumour necrosis factor-α receptor 1; hsCRP: High-sensitivity C-reactive protein; IL-6: Interleukin 6; eGFR: estimated glomerular filtration rate.
(DOCX)
