# Supplementary material for: Inflammation and Renal Function after a Four-Year Follow-Up in Subjects with Unimpaired Glomerular Filtration Rate: Results from the Observational, Population-Based CARLA Cohort
Source: PLoS One. 2014 Sep 26;9(9):e108427. doi: 10.1371/journal.pone.0108427 (PMC4178159; doi:10.1371/journal.pone.0108427)
Supplement: File S1 — Contains Table S1, Cross-sectional and longitudinal linear regression analysis in men: association of inflammation parameters with GFR/creatinine. Glomerular filtration rate estimated by means of MDRD formula. (effect estimates with 95% confidence intervals). unadj. = unadjusted estimates; adj. = estimates adjusted for age, body mass index (BMI), HbA1c, low-density lipoprotein (LDL), high-density lipoprotein (HDL), baseline diastolic and systolic blood pressure and baseline to follow-up change in the longitudinal analyses, number of cigarettes/cigars/pipes smoked, presence of cardio-vascular diseases, regular intake of anti-diabetic (Anatomical Therapeutic Chemical Classification [ATC]: A10) and anti-hypertensive medication (ATC: C02/C03/C07/C08/C09); users coded as “1”, non-users coded as “0”. Abbreviations: sTNF-R1: Soluble tumour necrosis factor-α receptor 1; hsCRP: High-sensitivity C-reactive protein; IL-6: Interleukin 6; eGFR: estimated glomerular filtration rate. Table S2, Cross-sectional and longitudinal linear regression analysis in women: association of inflammation parameters with GFR/creatinine. Glomerular filtration rate estimated by means of MDRD formula. (effect estimates with 95% confidence intervals). unadj. = unadjusted estimates; adj. = estimates adjusted for age, body mass index (BMI), HbA1c, low-density lipoprotein (LDL), high-density lipoprotein (HDL), baseline diastolic and systolic blood pressure and baseline to follow-up change in the longitudinal analyses, number of cigarettes/cigars/pipes smoked, presence of cardio-vascular diseases, regular intake of anti-diabetic (Anatomical Therapeutic Chemical Classification [ATC]: A10) and anti-hypertensive medication (ATC: C02/C03/C07/C08/C09); users coded as “1”, non-users coded as “0”. Abbreviations: sTNF-R1: Soluble tumour necrosis factor-α receptor 1; hsCRP: High-sensitivity C-reactive protein; IL-6: Interleukin 6; eGFR: estimated glomerular filtration rate. Table S3, Cross-sectional and longitudi [file pone.0108427.s001.docx]

Table S1: Cross-sectional and longitudinal linear regression analysis in men: association of inflammation parameters with GFR/creatinine. Glomerular filtration rate estimated by means of MDRD formula. (effect estimates with 95% confidence intervals)

| **Men** | |  |  |  |  |  | |  |
| --- | --- | --- | --- | --- | --- | --- | --- | --- |
| **Cross-sectional analysis** | | | | | | | | |
| **Continuous Outcome** | | eGFR (95% CI) | p | Partial correlation | Creatinine (95% CI) | | p | Partial correlation |
|  |  | [mL/min/1.73m^2^] |  | coefficient | [mmol/L] | |  | coefficient |
| sTNF-R1 (100 pg/mL) | unadj. | -1.5 [-1.7, -1.2] | <.0001 | 0.132 | 0.9 [0.8, 1.1] | | <.0001 | 0.124 |
|  | adj. | -1.0 [-1.3, -0.8] | <.0001 | 0.068 | 0.8 [0.6, 1.0] | | <.0001 | 0.084 |
| hsCRP (10 mg/L) | unadj. | -1.9 [-5.0, 1.1] | 0.2124 | 0.002 | 1.5 [-0.5, 3.5] | | 0.1321 | 0.003 |
|  | adj. | -2.1 [-4.9, 0.8] | 0.1522 | 0.003 | 1.7 [-0.3, 3.6] | | 0.0956 | 0.003 |
| IL-6 (10 pg/mL) | unadj. | 0.0 [-0.6, 0.7] | 0.9013 | <0.001 | 0.0 [-0.5, 0.4] | | 0.8447 | <0.001 |
|  | adj. | -0.1 [-0.7, 0.5] | 0.7971 | <0.001 | 0.0 [-0.4, 0.4] | | 0.9461 | <0.001 |
| **Longitudinal analysis (change in eGFR/Creatinine)** | | | | | | | | |
| **Continuous Outcome** |  | eGFR (95% CI) | p | Partial correlation | Creatinine (95% CI) | | p | Partial correlation |
|  |  | [mL/min/1.73m^2^] |  | coefficient | [mmol/L] | |  | coefficient |
| sTNF-R1 (100 pg/mL) | unadj. | -0.7 [-1.0, -0.4] | <.0001 | 0.034 | 1.2 [1.0, 1.5] | | <.0001 | 0.113 |
|  | adj. | -0.5 [-0.8, -0.2] | 0.0011 | 0.016 | 1.1 [0.8, 1.4] | | <.0001 | 0.077 |
| hsCRP (10 mg/L) | unadj. | -0.4 [-3.2, 2.5] | 0.8009 | <0.001 | 0.4 [-2.2, 3.0] | | 0.7547 | <0.001 |
|  | adj. | 0.0 [-2.9, 2.9] | 0.9946 | <0.001 | 0.3 [-2.3, 2.9] | | 0.8360 | <0.001 |
| IL-6 (10 pg/mL) | unadj. | -0.8 [-1.9, 0.3] | 0.1561 | 0.003 | 0.8 [-0.3, 1.9] | | 0.1410 | 0.003 |
|  | adj. | -0.6 [-1.7, 0.6] | 0.3252 | 0.002 | 0.6 [-0.5, 1.6] | | 0.2782 | 0.002 |

unadj.= unadjusted estimates; adj.=estimates adjusted for age, body mass index (BMI), HbA1c, low-density lipoprotein (LDL), high-density lipoprotein (HDL), baseline diastolic and systolic blood pressure and baseline to follow-up change in the longitudinal analyses, number of cigarettes/cigars/pipes smoked, presence of cardio-vascular diseases, regular intake of anti-diabetic (Anatomical Therapeutic Chemical Classification [ATC]: A10) and anti-hypertensive medication (ATC: C02/C03/C07/C08/C09) ; users coded as “1”, non-users coded as “0”.

Abbreviations: sTNF-R1: Soluble tumour necrosis factor-α receptor 1; hsCRP: High-sensitivity C-reactive protein; IL-6: Interleukin 6; eGFR: estimated glomerular filtration rate

Table S2: Cross-sectional and longitudinal linear regression analysis in women: association of inflammation parameters with GFR/creatinine. Glomerular filtration rate estimated by means of MDRD formula. (effect estimates with 95% confidence intervals)

| **Women** | |  |  |  |  |  | |  |
| --- | --- | --- | --- | --- | --- | --- | --- | --- |
| **Cross-sectional analysis** | | | | | | | | |
| **Continuous Outcome** | | eGFR (95% CI) | p | Partial correlation | Creatinine (95% CI) | | p | Partial correlation |
|  |  | [mL/min/1.73m^2^] |  | coefficient | [mmol/L] | |  | coefficient |
| sTNF-R1 (100 pg/mL) | unadj. | -1.3 [-1.6, -1.0] | <.0001 | 0.090 | 0.6 [0.5, 0.8] | | <.0001 | 0.076 |
|  | adj. | -0.9 [-1.3, -0.5] | <.0001 | 0.037 | 0.6 [0.4, 0.8] | | <.0001 | 0.049 |
| hsCRP (10 mg/L) | unadj. | -0.6 [-2.3, 1.2] | 0.5272 | 0.001 | 0.3 [-0.7, 1.2] | | 0.5771 | <0.001 |
|  | adj. | -1.3 [-5.3, 2.7] | 0.5235 | <0.001 | 1 [-1.2, 3.3] | | 0.3614 | <0.001 |
| IL-6 (10 pg/mL) | unadj. | 0.2 [-0.1, 0.5] | 0.2492 | 0.002 | -0.1 [-0.3, 0.1] | | 0.2812 | 0.002 |
|  | adj. | 0.2 [-0.1, 0.5] | 0.2829 | 0.002 | -0.1 [-0.3, 0.1] | | 0.2357 | 0.002 |
| **Longitudinal analysis (change in eGFR/Creatinine)** | | | | | | | | |
| **Continuous Outcome** |  | eGFR (95% CI) | p | Partial correlation | Creatinine (95% CI) | | p | Partial correlation |
|  |  | [mL/min/1.73m^2^] |  | coefficient | [mmol/L] | |  | coefficient |
| sTNF-R1 (100 pg/mL) | unadj. | -0.2 [-0.5, 0.1] | 0.2013 | 0.003 | 0.3 [0.1, 0.6] | | 0.0051 | 0.015 |
|  | adj. | -0.1 [-0.5, 0.2] | 0.5334 | 0.001 | 0.2 [-0.1, 0.5] | | 0.1778 | 0.003 |
| hsCRP (10 mg/L) | unadj. | -1.7 [-5.0, 1.6] | 0.3180 | 0.002 | 1.2 [-1.3, 3.7] | | 0.3413 | 0.002 |
|  | adj. | -1.2 [-4.7, 2.3] | 0.5002 | 0.001 | 1.3 [-1.3, 3.9] | | 0.3162 | 0.002 |
| IL-6 (10 pg/mL) | unadj. | -2.2 [-3.5, -0.5] | 0.0086 | 0.013 | 1.3 [0.0, 2.5] | | 0.0435 | 0.008 |
|  | adj. | -2.4 [-4.0, -0.7] | 0.0050 | 0.014 | 1.3 [0.0, 2.5] | | 0.0463 | 0.008 |

unadj.= unadjusted estimates; adj.=estimates adjusted for age, body mass index (BMI), HbA1c, low-density lipoprotein (LDL), high-density lipoprotein (HDL), baseline diastolic and systolic blood pressure and baseline to follow-up change in the longitudinal analyses, number of cigarettes/cigars/pipes smoked, presence of cardio-vascular diseases, regular intake of anti-diabetic (Anatomical Therapeutic Chemical Classification [ATC]: A10) and anti-hypertensive medication (ATC: C02/C03/C07/C08/C09) ; users coded as “1”, non-users coded as “0”.

Abbreviations: sTNF-R1: Soluble tumour necrosis factor-α receptor 1; hsCRP: High-sensitivity C-reactive protein; IL-6: Interleukin 6; eGFR: estimated glomerular filtration rate

Table S3: Cross-sectional and longitudinal linear regression analysis in men: association of inflammation parameters with GFR/creatinine after exclusion of subjects older than 71 years at baseline. Glomerular filtration rate estimated by means of CKD-EPI formula. (effect estimates with 95% confidence intervals)

| **Men** | |  |  |  |  |  | |  |
| --- | --- | --- | --- | --- | --- | --- | --- | --- |
| **Cross-sectional analysis** | | | | | | | | |
| **Continuous Outcome** | | eGFR (95% CI) | p | Partial correlation | Creatinine (95% CI) | | p | Partial correlation |
|  |  | [mL/min/1.73m^2^] |  | coefficient | [mmol/L] | |  | coefficient |
| sTNF-R1 (100 pg/mL) | unadj. | -0.8 [-1, -0.6] | <.0001 | 0.080 | 0.7 [0.4, 0.9] | | <.0001 | 0.053 |
|  | adj. | -0.6 [-0.8, -0.4] | <.0001 | 0.052 | 0.7 [0.5, 1.0] | | <.0001 | 0.056 |
| hsCRP (10 mg/L) | unadj. | -1.5 [-3.6, 0.5] | 0.1363 | 0.004 | 1.5 [-0.6, 3.6] | | 0.1604 | 0.003 |
|  | adj. | -1.7 [-3.5, 0.0] | 0.0484 | 0.007 | 2.3 [-0.2, 4.4] | | 0.0353 | 0.008 |
| IL-6 (10 pg/mL) | unadj. | 0.1 [-0.3, 0.6] | 0.5185 | 0.001 | 0.0 [-0.4, 0.5] | | 0.8611 | <0.001 |
|  | adj. | 0.0 [-0.4, 0.3] | 0.8379 | <0.001 | 0.1 [-0.3, 0.5] | | 0.6685 | <0.001 |
| **Longitudinal analysis (change in eGFR/Creatinine)** | | | | | | | | |
| **Continuous Outcome** |  | eGFR (95% CI) | p | Partial correlation | Creatinine (95% CI) | | p | Partial correlation |
|  |  | [mL/min/1.73m^2^] |  | coefficient | [mmol/L] | |  | coefficient |
| sTNF-R1 (100 pg/mL) | unadj. | -0.8 [-1, -0.6] | <.0001 | 0.094 | 1.4 [1.1, 1.7] | | <.0001 | 0.136 |
|  | adj. | -0.7 [-1, -0.5] | <.0001 | 0.068 | 1.2 [0.9, 1.5] | | <.0001 | 0.105 |
| hsCRP (10 mg/L) | unadj. | -0.9 [-2.8, 0.9] | 0.3315 | 0.002 | 0.8 [-1.5, 3.2] | | 0.4878 | 0.001 |
|  | adj. | -0.6 [-2.5, 1.3] | 0.5553 | 0.001 | 0.5 [-1.9, 2.9] | | 0.6796 | <0.001 |
| IL-6 (10 pg/mL) | unadj. | -0.7 [-1.8, 0.4] | 0.1927 | 0.003 | 1.4 [-0.1, 2.9] | | 0.0762 | 0.006 |
|  | adj. | -0.5 [-1.6, 0.6] | 0.3475 | 0.003 | 1.1 [-0.3, 2.6] | | 0.1328 | 0.006 |

unadj.= unadjusted estimates; adj.=estimates adjusted for age, body mass index (BMI), HbA1c, low-density lipoprotein (LDL), high-density lipoprotein (HDL), baseline diastolic and systolic blood pressure and baseline to follow-up change in the longitudinal analyses, number of cigarettes/cigars/pipes smoked, presence of cardio-vascular diseases, regular intake of anti-diabetic (Anatomical Therapeutic Chemical Classification [ATC]: A10) and anti-hypertensive medication (ATC: C02/C03/C07/C08/C09) ; users coded as “1”, non-users coded as “0”.

Abbreviations: sTNF-R1: Soluble tumour necrosis factor-α receptor 1; hsCRP: High-sensitivity C-reactive protein; IL-6: Interleukin 6; eGFR: estimated glomerular filtration rate

Table S4: Cross-sectional and longitudinal linear regression analysis in women: association of inflammation parameters with GFR/creatinine after exclusion of subjects older than 71 years at baseline. Glomerular filtration rate estimated by means of CKD-EPI formula. (effect estimates with 95% confidence intervals)

| **Women** | |  |  |  |  |  | |  |
| --- | --- | --- | --- | --- | --- | --- | --- | --- |
| **Cross-sectional analysis** | | | | | | | | |
| **Continuous Outcome** | | eGFR (95% CI) | p | Partial correlation | Creatinine (95% CI) | | p | Partial correlation |
|  |  | [mL/min/1.73m^2^] |  | coefficient | [mmol/L] | |  | coefficient |
| sTNF-R1 (100 pg/mL) | unadj. | -1.0 [-1.2, -0.7] | <.0001 | 0.096 | 0.6 [0.4, 0.8] | | <.0001 | 0.050 |
|  | adj. | -0.6 [-0.9, -0.4] | <.0001 | 0.041 | 0.6 [0.3, 0.8] | | <.0001 | 0.039 |
| hsCRP (10 mg/L) | unadj. | 1.3 [-1.1, 3.6] | 0.3001 | 0.002 | -1.2 [-3.1, 0.8] | | 0.2336 | 0.003 |
|  | adj. | 1.3 [-1.0, 3.5] | 0.2264 | 0.002 | -1.3 [-3.2, 0.8] | | 0.2326 | 0.003 |
| IL-6 (10 pg/mL) | unadj. | 0.1 [-0.1, 0.4] | 0.1774 | 0.004 | -0.1 [-0.3, 0.1] | | 0.2981 | 0.002 |
|  | adj. | 0.1 [-0.1, 0.3] | 0.3168 | 0.002 | -0.1 [-0.3, 0.1] | | 0.3024 | 0.002 |
| **Longitudinal analysis (change in eGFR/creatinie)** | | | | | | | | |
| **Continuous Outcome** |  | eGFR (95% CI) | p | Partial correlation | Creatinine (95% CI) | | p | Partial correlation |
|  |  | [mL/min/1.73m^2^] |  | coefficient | [mmol/L] | |  | coefficient |
| sTNF-R1 (100 pg/mL) | unadj. | 0.0 [-0.3, 0.2] | 0.8743 | <0.001 | 0.1 [-0.2, 0.3] | | 0.4619 | 0.001 |
|  | adj. | 0.1 [-0.2, 0.3] | 0.7139 | <0.001 | 0.0 [-0.3, 0.3] | | 0.9891 | <0.001 |
| hsCRP (10 mg/L) | unadj. | -0.4 [-3.0, 2.2] | 0.7490 | <0.001 | 0.8 [-1.8, 3.3] | | 0.5535 | 0.001 |
|  | adj. | 0.4 [-2.4, 3.1] | 0.7949 | <0.001 | -0.1 [-2.8, 2.7] | | 0.9639 | <0.001 |
| IL-6 (10 pg/mL) | unadj. | -1.4 [-2.5, -0.2] | 0.0221 | 0.011 | 1.3 [0.1, 2.4] | | 0.0405 | 0.010 |
|  | adj. | -1.5 [-2.7, -0.3] | 0.0163 | 0.011 | 1.3 [0.1, 2.5] | | 0.0362 | 0.010 |

unadj.= unadjusted estimates; adj.=estimates adjusted for age, body mass index (BMI), HbA1c, low-density lipoprotein (LDL), high-density lipoprotein (HDL), baseline diastolic and systolic blood pressure and baseline to follow-up change in the longitudinal analyses, number of cigarettes/cigars/pipes smoked, presence of cardio-vascular diseases, regular intake of anti-diabetic (Anatomical Therapeutic Chemical Classification [ATC]: A10) and anti-hypertensive medication (ATC: C02/C03/C07/C08/C09) ; users coded as “1”, non-users coded as “0”.

Abbreviations: sTNF-R1: Soluble tumour necrosis factor-α receptor 1; hsCRP: High-sensitivity C-reactive protein; IL-6: Interleukin 6; eGFR: estimated glomerular filtration rate
